# Supplementary figures and images for: Adaptive divergence, neutral panmixia, and algal symbiont population structure in the temperate coral Astrangia poculata along the Mid-Atlantic United States
Source: PeerJ. 2020 Nov 18;8:e10201. doi: 10.7717/peerj.10201 (PMC7680023; doi:10.7717/peerj.10201)

A. Mean Temperature

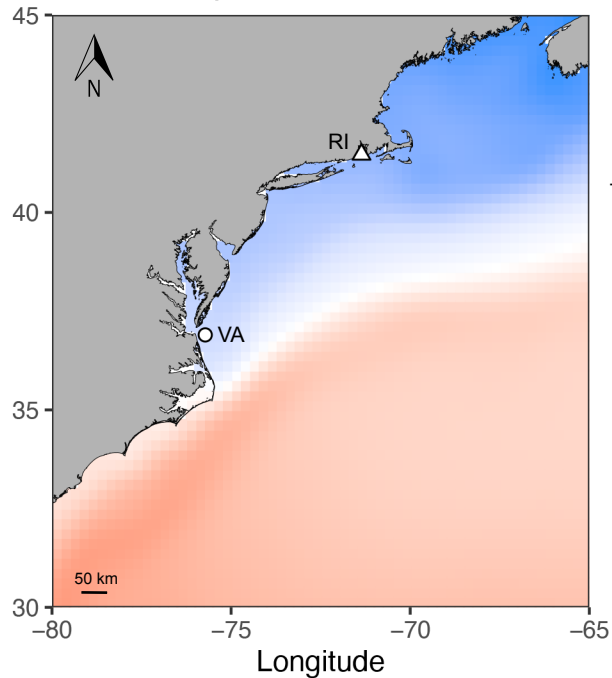

B. Minimum Temperature

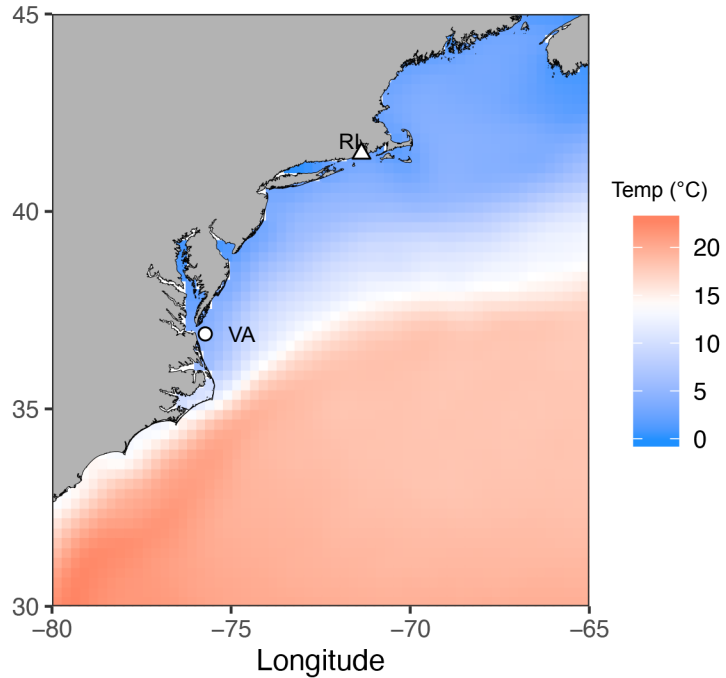

Supplement: Supplemental Information 1 — Virginia = VA and Rhode Island = RI. The over-water distance between the two collection sites is approximately 630 km. [file peerj-08-10201-s001.pdf]

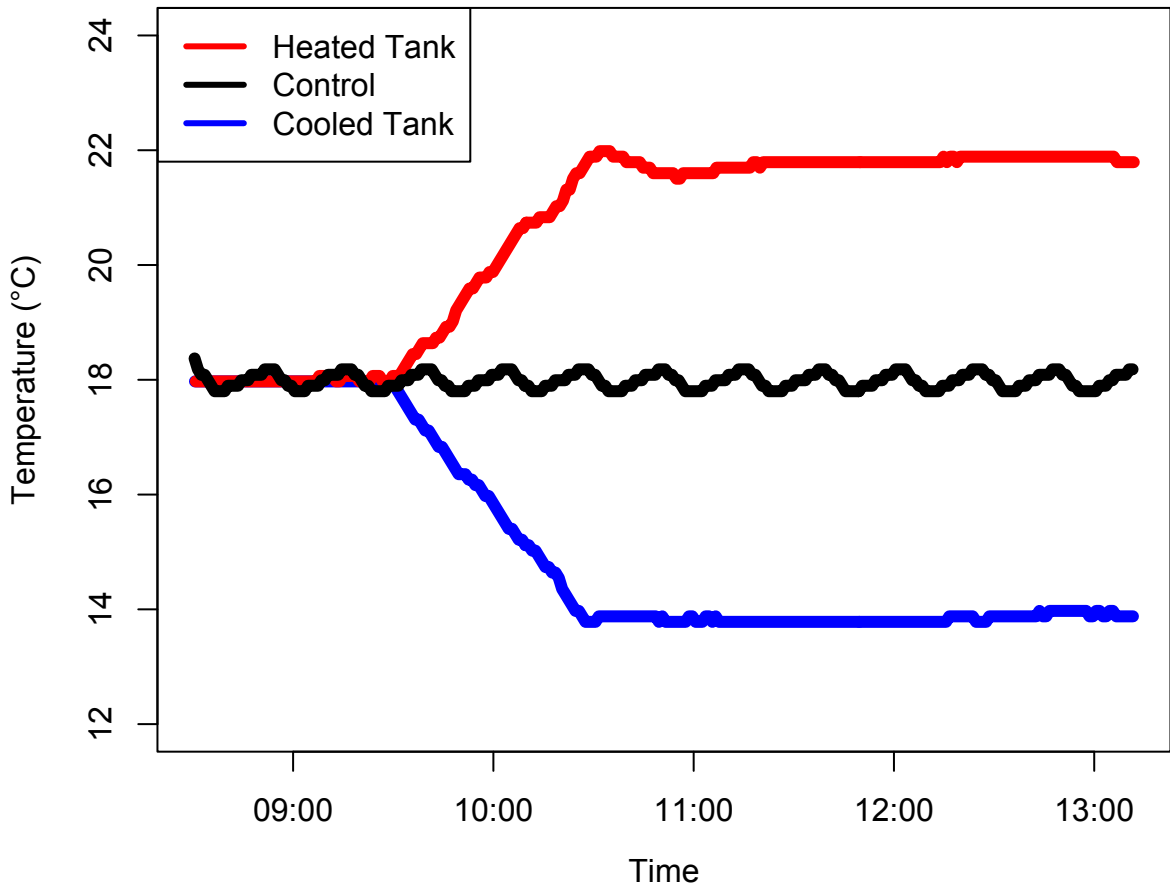

Supplement: Supplemental Information 2 — Temperature was recorded using Hobo Pendant® Temperature Data Loggers (Onset Computer Corporation, Bourne, MA, USA). Sampling for RNA occurred between 11:30 and 13:10. [file peerj-08-10201-s002.pdf]

# Cluster Dendrogram

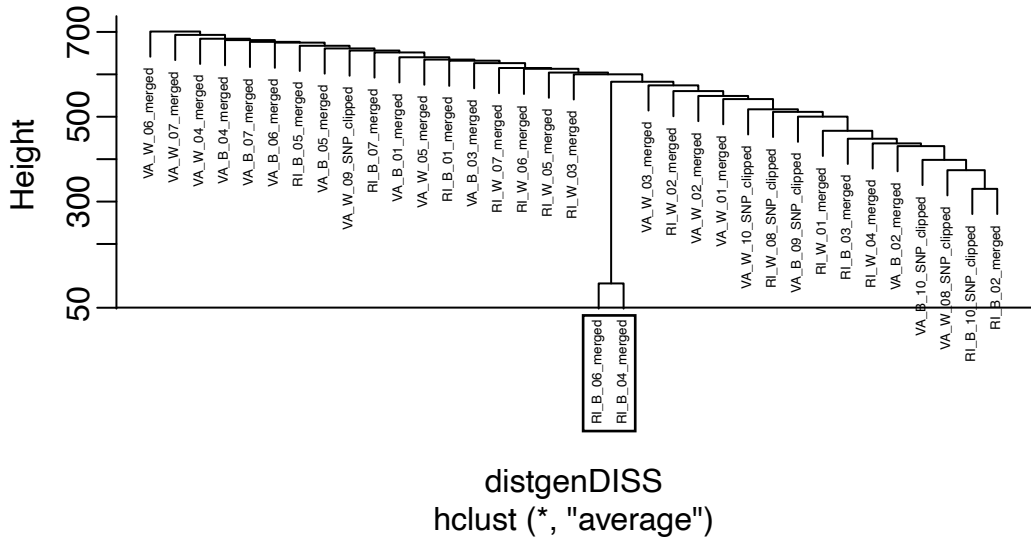

Supplement: Supplemental Information 3 — Height on the y-axis is based on Hamming’s distance, or the number of allelic differences between individuals. The box indicates the two putative clones that had relatively few allelic differences, and RI_B_06_merged was removed for all downstream analyses. [file peerj-08-10201-s003.pdf]

## A. Coral Host Neutral Loci

K = 2

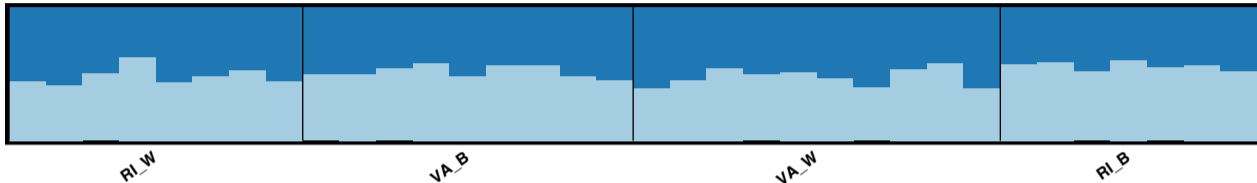

## B. Coral Host Outlier Loci

K = 2

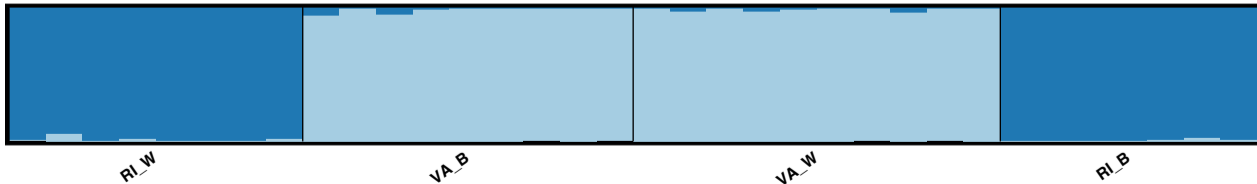

Supplement: Supplemental Information 4 — Vertical columns represent individual samples within a population and the associated probability of assignment to K = 2 genetic clusters (light blue and dark blue). The four site abbreviations are: VA-B (brown/symbiotic Virginia), VA-W (white/aposymbiotic Virginia), RI-B (brown/symbiotic Rhode Island), and RI-W (white/aposymbiotic Rhode Island). [file peerj-08-10201-s004.pdf]

## A. Molecular Functions

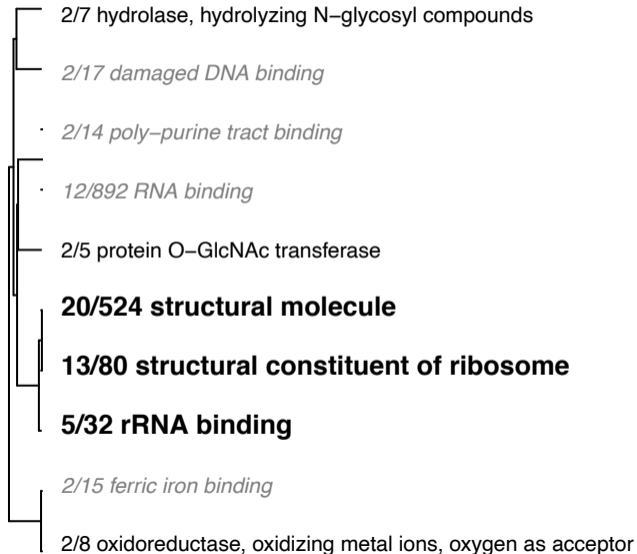

## B. Cellular Components

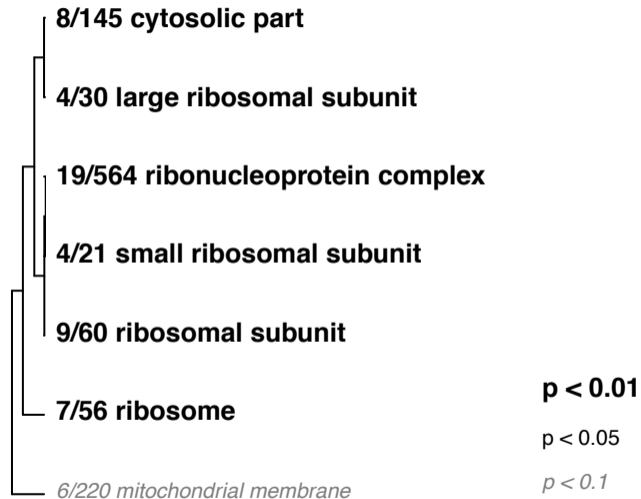

Supplement: Supplemental Information 5 — Dendrograms indicate gene sharing between GO categories. The numbers listed with each GO term correspond to the number of genes in the module over the total number of genes assigned to each category in the entire SNP dataset. [file peerj-08-10201-s005.pdf]
