# Supplementary material for: Adaptive divergence, neutral panmixia, and algal symbiont population structure in the temperate coral Astrangia poculata along the Mid-Atlantic United States
Source: PeerJ. 2020 Nov 18;8:e10201. doi: 10.7717/peerj.10201 (PMC7680023; doi:10.7717/peerj.10201)
Supplement: Supplemental Information 6 — The sequences were added to one of three databases (‘Database’): 1) Dirty Coral = all available cnidarian data, 2) Clean Coral = aposymbiotic cnidarian data only, 3) Clean Sym = cultured Symbiodiniaceae data only. ‘Reference’ refers to the species the sequencing data was obtained from and ‘Source’ refers to the publication or website associated with the data. [file peerj-08-10201-s006.docx]

| Reference | Database | Source |
| --- | --- | --- |
| *Edwardsiella lineata* | Dirty Coral | Stefanik et al. (2014) |
| Favia sp. | Clean Coral | Mehr et al. (2013) |
| *Pocillopora damicornis* | Dirty Coral | Traylor-Knowles et al. (2011) |
| *Nematostella vectensis* | Clean Coral | Putnam et al. (2007) |
| Clade-B1 (*Breviolum minutum*) | Clean Sym | Shoguchi et al. (2013) |
| Cultured Symbiodiniaceae,  15 species | Clean Sym | Zhang et al. (2007) |
| Cultured free-living Symbiodiniaceae, strains: CCMP421, CCMP2430, MMETSP1122, MMETSP1123 | Clean Sym | Imicrobe  http://mirrors.iplantcollaborative.org/browse/iplant/home/shared/imicrobe/projects/104/transcriptomes/MMETSP111/ |
| *Acropora digitifera* sperm | Clean Coral | Shinzato et al. (2011) |
| *Acropora palmata* eggs | Clean Coral | Schwarz et al. (2008) |
| *Montastrea faveolata* eggs | Clean Coral | Schwarz et al. (2008) |
| *Hydra vulgaris* | Clean Coral | Chapman et al. (2010) |
| *Mnemiopsis leidyi* | Clean Coral | Ryan et al. (2013) |
| *Monosiga brevicollis* | Clean Sym | King et al. (2008) |
| *Pleurobranchia pileus* | Clean Coral | Moroz et al. (2014) |
| *Platygyra carnosus* | Dirty Coral | Sun et al. (2013) |
| *Gorgonia ventalina* | Dirty Coral | Burge et al. (2013) |

Burge CA, Mouchka ME, Harvell CD, and Roberts S. 2013. Immune response of the Caribbean sea fan, Gorgonia ventalina, exposed to an Aplanochytrium parasite as revealed by transcriptome sequencing. *Frontiers in Physiology* 4:180.

Chapman JA, Kirkness EF, Simakov O, Hampson SE, Mitros T, Weinmaier T, Rattei T, Balasubramanian PG, Borman J, and Busam D. 2010. The dynamic genome of Hydra. *Nature* 464:592.

Davies SW, Marchetti A, Ries JB, and Castillo KD. 2016. Thermal and *p*CO2 stress elicit divergent transcriptomic responses in a resilient coral. *Frontiers in Marine Science* 3:112.

Mehr SFP, DeSalle R, Kao H-T, Narechania A, Han Z, Tchernov D, Pieribone V, and Gruber DF. 2013. Transcriptome deep-sequencing and clustering of expressed isoforms from Favia corals. *BMC Genomics* 14:546.

Moroz LL, Kocot KM, Citarella MR, Dosung S, Norekian TP, Povolotskaya IS, Grigorenko AP, Dailey C, Berezikov E, and Buckley KM. 2014. The ctenophore genome and the evolutionary origins of neural systems. *Nature* 510:109.

Putnam NH, Srivastava M, Hellsten U, Dirks B, Chapman J, Salamov A, Terry A, Shapiro H, Lindquist E, and Kapitonov VV. 2007. Sea anemone genome reveals ancestral eumetazoan gene repertoire and genomic organization. *Science* 317:86-94.

Ryan JF, Pang K, Schnitzler CE, Nguyen A-D, Moreland RT, Simmons DK, Koch BJ, Francis WR, Havlak P, and Smith SA. 2013. The genome of the ctenophore Mnemiopsis leidyi and its implications for cell type evolution. *Science* 342:1242592.

Schwarz JA, Brokstein PB, Voolstra C, Terry AY, Miller DJ, Szmant AM, Coffroth MA, and Medina M. 2008. Coral life history and symbiosis: functional genomic resources for two reef building Caribbean corals, Acropora palmata and Montastraea faveolata. *BMC Genomics* 9:97.

Shinzato C, Shoguchi E, Kawashima T, Hamada M, Hisata K, Tanaka M, Fujie M, Fujiwara M, Koyanagi R, and Ikuta T. 2011. Using the Acropora digitifera genome to understand coral responses to environmental change. *Nature* 476:320.

Shoguchi E, Shinzato C, Kawashima T, Gyoja F, Mungpakdee S, Koyanagi R, Takeuchi T, Hisata K, Tanaka M, and Fujiwara M. 2013. Draft assembly of the Symbiodinium minutum nuclear genome reveals dinoflagellate gene structure. *Current Biology* 23:1399-1408.

Stefanik DJ, Lubinski TJ, Granger BR, Byrd AL, Reitzel AM, DeFilippo L, Lorenc A, and Finnerty JR. 2014. Production of a reference transcriptome and transcriptomic database (EdwardsiellaBase) for the lined sea anemone, Edwardsiella lineata, a parasitic cnidarian. *BMC Genomics* 15:71.

Sun J, Chen Q, Lun JC, Xu J, and Qiu J-W. 2013. PcarnBase: development of a transcriptomic database for the brain coral Platygyra carnosus. *Marine Biotechnology* 15:244-251.

Traylor-Knowles N, Granger BR, Lubinski TJ, Parikh JR, Garamszegi S, Xia Y, Marto JA, Kaufman L, and Finnerty JR. 2011. Production of a reference transcriptome and transcriptomic database (PocilloporaBase) for the cauliflower coral, Pocillopora damicornis. *BMC Genomics* 12:585.

Zhang H, Hou Y, Miranda L, Campbell DA, Sturm NR, Gaasterland T, and Lin S. 2007. Spliced leader RNA trans-splicing in dinoflagellates. *Proceedings of the National Academy of Sciences* 104:4618-4623.
