# Supplementary material for: Adaptive divergence, neutral panmixia, and algal symbiont population structure in the temperate coral Astrangia poculata along the Mid-Atlantic United States
Source: PeerJ. 2020 Nov 18;8:e10201. doi: 10.7717/peerj.10201 (PMC7680023; doi:10.7717/peerj.10201)
Supplement: Supplemental Information 7 — Data is included for the putatively neutral coral SNPs (A, n = 279), high outlier coral SNPs (B,n = 66), neutral symbiont SNPs (C, n = 20) and high outlier symbiont SNPs (D, n = 4). Observed heterozygosity (Ho) is the observed frequency of heterozygotes within a subpopulation, values can range from 0 (where all individuals are homozygous) to 1 (where all individuals are heterozygous). Heterozygosity within populations (or expected heterozygosity, Hs) is the expected frequency of heterozygotes within subpopulations (assuming Hardy-Weinberg equilibrium) and includes a correction for sampling bias from sampling a limited number of individuals per population. Values were calculated in Genodive using default parameters. [file peerj-08-10201-s007.docx]

| Population | Observed Heterozygosity (Ho) | Expected Heterozygosity (Hs) |
| --- | --- | --- |
| *A. Neutral Coral* |  |  |
| VA-B | 0.228 | 0.248 |
| VA-W | 0.238 | 0.267 |
| RI-B | 0.217 | 0.224 |
| RI-W | 0.283 | 0.257 |
| *B. High Outlier Coral* |  |  |
| VA-B | 0.121 | 0.168 |
| VA-W | 0.097 | 0.171 |
| RI-B | 0.191 | 0.324 |
| RI-W | 0.292 | 0.283 |
| *C. Neutral Symbiont* |  |  |
| VA-B | 0.575 | 0.385 |
| RI-B | 0.47 | 0.338 |
| *D. High Outlier Symbiont* |  |  |
| VA-B | 0.000 | 0.000 |
| RI-B | 0.050 | 0.047 |
