# Supplementary material for: Adaptive divergence, neutral panmixia, and algal symbiont population structure in the temperate coral Astrangia poculata along the Mid-Atlantic United States
Source: PeerJ. 2020 Nov 18;8:e10201. doi: 10.7717/peerj.10201 (PMC7680023; doi:10.7717/peerj.10201)
Supplement: Supplemental Information 8 — Aand Binclude contigs with more than one outlier SNP from the Astrangia poculata coral host and Breviolum psygmophilum symbiont analyses, respectively. Cincludes information on the contigs that were shared across independent Virginia (VA) and Rhode Island (RI) analyses used to look for signatures of genetic differentiation by symbiotic state. ‘Contig Name’ and ‘Contig Length’ refers to the name and length of the contig containing the SNP(s) in the reference transcriptome. The description of the contigs was determined by blasting to the NCBI nr database. The E-value and percent match describe the match between the reference transcriptome contig and the nr database match. ‘No Description’ and ‘No sig hit’ refer to contigs that did not match an annotation. [file peerj-08-10201-s008.docx]

| # SNPs on Contig | Contig Name | Contig Length | Description | E-value | Percent Match |
| --- | --- | --- | --- | --- | --- |
| *A. Coral Host, multiple SNPs per contig* | | | | | |
| 2 | TR6468\|c0_g1_i2_coral | 552 | 40S ribosomal protein S3 | 2.85E-126 | 0.98 |
| 2 | TR13823\|c0_g3_i2_coral | 863 | Copper-transporting ATPase 2 | 7.59E-153 | 0.89 |
| 2 | TR43764\|c4_g1_i1_coral | 2006 | Angiopoietin-1 receptor | 0 | 0.73 |
| 2 | TR43821\|c0_g1_i1_coral | 2019 | Apolipoprotein B-100 | 0 | 0.71 |
| 2 | TR47922\|c2_g2_i1_coral | 1120 | No Description | No sig hit | No sig hit |
| 2 | TR47926\|c0_g3_i1_coral | 1402 | Protein FEV | 2.05E-136 | 0.97 |
| 2 | TR47928\|c0_g1_i1_coral | 4713 | Collagen alpha-1(II) chain | 0 | 0.84 |
| 2 | TR47957\|c0_g1_i1_coral | 1549 | Adenosylhomocysteinase | 0 | 0.94 |
| 3 | TR47983\|c0_g2_i1_coral | 2074 | Collagen alpha chain | 4.26E-164 | 0.85 |
| 4 | TR48002\|c0_g2_i1_coral | 2098 | Myosin heavy chain, muscle | 0 | 0.89 |
| 2 | TR52526\|c0_g1_i1_coral | 1204 | Cyclic AMP-dependent transcription factor ATF-5 | 1.74E-141 | 0.74 |
| 2 | TR52571\|c0_g1_i1_coral | 552 | Ubiquitin-like protein FUBI | 1.13E-74 | 0.87 |
| 3 | TR52932\|c0_g1_i1_coral | 531 | 60S ribosomal protein L26 | 3.52E-73 | 0.96 |
| *B. Symbiont Outlier SNPs* | | | | | |
| 1 | TR17944\|c3_g1_i1_sym | 1612 | Photosystem II CP43 reaction center protein | 0 | 0.94 |
| 1 | TR28648\|c0_g2_i1_sym | 732 | Photosystem I P700 chlorophyll *a* apoprotein A2 | 5.53E-158 | 0.9 |
| 1 | TR41328\|c0_g1_i1_sym | 599 | Photosystem II protein D1 | 3.46E-106 | 0.98 |
| 1 | TR47986\|c1_g3_i2_sym | 761 | No Description | No sig hit | No sig hit |
| *C. Outlier SNPs Shared by Independent VA and RI Analysis* | | | | | |
| 3 | TR6468\|c0_g1_i1_coral | 839 | 40S ribosomal protein S3 | 2.25E-160 | 0.98 |
| 2 | TR6468\|c0_g1_i2_coral | 552 | 40S ribosomal protein S3 | 2.85E-126 | 0.98 |
| 1 | TR8375\|c0_g1_i1_coral | 748 | 60S ribosomal protein L12 | 1.60E-98 | 0.96 |
| 2 | TR13816\|c0_g1_i1_coral | 1306 | Sequestosome-1 | 9.40E-88 | 0.85 |
| 1 | TR20421\|c4_g1_i1_coral | 592 | No Description | No sig hit | No sig hit |
| 1 | TR47963\|c0_g2_i2_coral | 2536 | Collagen alpha-1(II) chain | 7.75E-49 | 0.54 |
| 1 | TR52526\|c0_g1_i1_coral | 1204 | Cyclic AMP-dependent transcription factor ATF-5 | 1.74E-141 | 0.74 |
